# Associations of antenatal maternal psychological distress with infant birth and development outcomes: Results from a South African birth cohort

**DOI:** 10.1016/j.comppsych.2019.152128

**Published:** 2020-01

**Authors:** RP MacGinty, SM Kariuki, W Barnett, CJ Wedderburn, A Hardy, N Hoffman, CR Newton, HJ Zar, KA Donald, DJ Stein

**Affiliations:** a Department of Paediatrics and Child Health, Red Cross War Memorial Children’s Hospital, University of Cape Town, South Africa; b South African Medical Research Council (SAMRC), Unit on Child & Adolescent Health, Cape Town, South Africa; c KEMRI-Wellcome Trust Research Programme, Kilifi, Kenya; d Department of Psychiatry, University of Oxford, UK; e Department of Clinical Research, London School of Hygiene & Tropical Medicine, London, UK; f Statistical Consulting Service, Department of Statistical Science, University of Cape Town, South Africa; g Department of Psychiatry & Neuroscience Institute, University of Cape Town, South Africa; h South African Medical Research Council (SAMRC) Unit on Risk & Resilience in Mental Disorders, South Africa

**Keywords:** DCHS, drakenstein child health study, HIC, high income country, LMIC, low- and middle-income country, SRQ20, self-reporting questionnaire 20-item, BDI, beck depression inventory, IPV, intimate partner violence, PTSD, post-traumatic stress disorder, BSID III, bayley III scales of infant and toddler development, WAZ, weight for age z-score, HCAZ, head circumference for age z-score, SGA, small for gestational-age, AGA, appropriate for gestational-age, LGA, large for gestational-age, CI, confidence interval, Antenatal maternal psychological distress, Foetal growth, South Africa

## Abstract

**Background:**

Antenatal maternal psychological distress is common in low and middle-income countries (LMIC), but there is a dearth of research on its effect on birth and developmental outcomes in these settings, particularly in Sub-Saharan Africa. This study set out to identify risk factors for antenatal maternal psychological distress and determine whether antenatal maternal psychological distress was associated with infant birth and developmental outcomes, using data from the Drakenstein Child Health Study (DCHS), a birth cohort study in South Africa.

**Methods:**

Pregnant women were enrolled in the DCHS from primary care antenatal clinics. Antenatal maternal psychological distress was measured using the Self-Reporting Questionnaire 20-item (SRQ-20). A range of psychosocial measures, including maternal childhood trauma, depression, and posttraumatic stress disorder (PTSD) were administered. Birth outcomes, including premature birth, weight-for-age z-score and head circumference-for-age z-score, were measured using revised Fenton growth charts. The Bayley III Scales of Infant and Toddler Development was administered at 6 months of age to assess infant development outcomes, including cognitive, language, and motor domains in a subset of n = 231. Associations of maternal antenatal psychological distress with psychosocial measures, and with infant birth and developmental outcomes were examined using linear regression models.

**Results:**

961 women were included in this analysis, with 197 (21%) reporting scores indicating the presence of psychological distress. Antenatal psychological distress was associated with maternal childhood trauma, antenatal depression, and PTSD, and inversely associated with partner support. No association was observed between antenatal maternal psychological distress and preterm birth or early developmental outcomes, but antenatal maternal psychological distress was associated with a smaller head circumference at birth (coefficient=−0.30, 95% CI: −0.49; −0.10).

**Conclusion:**

Antenatal maternal psychological distress is common in LMIC settings and was found to be associated with key psychosocial measures during pregnancy, as well as with adverse birth outcomes, in our study population. These associations highlight the potential value of screening for antenatal maternal psychological distress as well as of developing targeted interventions.

## Highlights

• High levels of antenatal maternal psychological distress in a South African context.
• Maternal childhood trauma, PTSD and depression linked with psychological distress.
• Antenatal maternal psychological distress found to predict lower birth weight.
• Antenatal psychological distress associated with smaller birth head circumference.

## 1 Introduction

Psychological distress, refers to a heterogenous range of symptoms, including anxiety, anguish, depression, and demoralisation [1,2]. When such symptoms are more severe, they may meet diagnostic criteria for major depression or an anxiety disorder. Maternal psychological distress is highly prevalent during the perinatal (pregnancy and postpartum) period, where approximately 13–25% of women in high income countries (HIC) are reported to suffer from symptoms of psychological distress [3,4]. Maternal psychological distress may be even more common in low and middle-income countries (LMIC), where there are a range of risk factors for these symptoms and disorders, including underlying socio-economic stressors [5].

A systematic review of maternal well-being in Africa [10] reported that the prevalence of poor mental/psychological health during pregnancy ranged between 12.5%–30.2% in six studies conducted in three countries, including Nigeria [[6], [7], [8]], Uganda [9,10] and Zimbabwe [11,12]. The prevalence of antenatal depression ranged from 4.3% to 17.4%, with a weighted mean prevalence of 11.3% (95% CI: 9.5%–13.1%) in five reviewed studies conducted in Nigeria, Morocco, and The Gambia [12]. Two more recent studies from Nigeria found a weighted mean prevalence of maternal anxiety during pregnancy of 14.8% (95% CI: 12.3%–17.4%) [12]. These studies suggest that the prevalence of maternal depression and anxiety disorders are high in Sub-Saharan Africa.

Risk factors that have previously been found to predict maternal depression and anxiety in HIC include poor marital relationships, history of psychological disorders, poor social support and stressful life events [[12], [13], [14], [15]]. In the limited number of African studies – including in Nigeria and Morocco – that have investigated risk factors associated with antenatal depression and anxiety, the most consistent risk factor identified was poor family and partner support [[16], [17], [18]]. None of these studies appeared to investigate stressful life events, such as maternal childhood trauma, intimate partner violence (IPV) exposure, or post-traumatic stress disorder (PTSD) as potential risk factors, which we hypothesised would be closely related to antenatal maternal psychological distress.

The impact of antenatal maternal psychological distress on infant birth outcomes has been researched in HIC. A number of studies have found no relation between antenatal maternal psychological distress and infant birth outcomes [[19], [20], [21]]; however, others have reported that antenatal maternal psychological distress contributed to negative birth outcomes, such as preterm birth, low birth weight and smaller head circumference [[22], [23], [24], [25], [26], [27], [28]]. Premature birth and low birth weight are associated with significant mortality worldwide, most of which occurs in LMIC [5,29]. In LMIC settings, including Brazil and Bangladesh, antenatal maternal distress was found to predict low birth weight and premature birth [30,31]. However, few studies of this nature have been conducted in an African setting. The Perinatal Maternal Mental Disorder in Ethiopia (P-MaMiE) Study, conducted in rural Ethiopia found no association between common mental disorders (CMD) or psychological distress, as measured by the SRQ-20 questionnaire, and lower birth weight, stillbirth or neonatal mortality [32]. P-MaMiE authors speculated that the lack of association could be due other environmental risk factors such as maternal undernutrition and poor socio-economic status that suppressed the effect of CMD on birth weight [32].

There is concern that antenatal maternal psychological distress may also have an impact on infant developmental outcomes that impact later health and education potential. Many studies have reported an association between antenatal maternal psychological distress and cognitive and behavioural development [[33], [34], [35], [36], [37], [38], [39]]; however, there have been conflicting results produced [40], with few studies from sub-Saharan Africa. In the P-MaMiE Study, investigators found no association between maternal CMD and developmental outcomes (cognitive, motor and language domains) at 12 months of age. The investigators speculated that delay in development may be due to continuous exposure to environmental risk factors rather than early exposure to maternal psychosocial risk factors [41]. Of note, maternal physical intimate partner violence (IPV) exposure was found to be associated with infant cognitive delay [41].

While there is research on the effects of antenatal maternal psychological distress on the infant in HIC, data are lacking in a LMIC context, particularly in Sub-Saharan Africa where increased prevalence of antenatal maternal psychological distress [12,42] and adverse infant birth and developmental outcomes occur [43]. The Drakenstein Child Health Study (DCHS), a multidisciplinary birth cohort investigating the determinants of child health in South Africa [44], previously found that antenatal depression had an adverse impact on birth outcomes [45]. Based on these findings, we hypothesised that antenatal maternal psychological distress would have a similar impact. In addition, we expanded on the previous study by considering the impact of antenatal maternal psychological distress on child development outcomes at 6 months of age.

The aim of this study was to explore the risk factors for antenatal maternal psychological distress and determine any associations between antenatal maternal psychological distress and infant birth and developmental outcomes in a South African birth cohort.

## 2 Methods

### 2.1 Participants

Pregnant women, 18 years or older, between 20 and 28 weeks of gestation and attending one of two primary health care clinics in the Drakenstein peri-urban sub-district of the Western Cape, South Africa were recruited in the Drakenstein Child Health Study (DCHS) [42,45]. The primary health care facilities included Mbekweni, serving predominately a population of black African ancestry and TC Newman, serving a mixed-ancestry population. The Drakenstein region consists largely of a low socioeconomic status (SES) community, which has a free primary health care system, that includes antenatal and child health services. All the women enrolled in the study provided written informed consent prior to participation. In addition, the DCHS was approved by the human research ethics committees (HREC) of the University of Cape Town and Stellenbosch University (HREC Reference number: 401/2009).

### 2.2 Measures

#### 2.2.1 Sociodemographic measures

Sociodemographic information was collected from the mother at enrolment, and many of these measures were adapted from items used in the South African Stress and Health Study [46]. This included a composite SES score that was used to categorise study participants into quartiles (low, low-moderate, moderate-high or high SES). Current employment, standardised scores of educational achievement, household income and an asset index score, were used to calculate the composite SES score [45]. It should be noted that these quartiles only relate to within-community comparisons and are not referenced to any external measure of SES.

#### 2.2.2 Maternal psychological distress

The Self-Reporting Questionnaire 20-item (SRQ-20) was used to assess maternal antenatal psychological distress. This is a reliable tool that can be used as a screen for mental disorders such as depression and anxiety disorders, and has been widely used in HIC and South African settings [[47], [48], [49], [50], [51]]. The questionaire was administered between 28 and 32 weeks of gestation in either English, Afrikaans or isiXhosa based on the preferred language of the participant [52]. Each item was assessed on a Yes-No dichotomous scale and a total score was obtained by summing responses across all 20 items. Example questions from the SRQ-20 in English include: “Do you feel unhappy?”; “Do you feel nervous, tense or worried” “Have you lost interest in things?” Based on prior literature, a cut-off score of ≥8 was used to define the threshold for psychological distress [42,47,48,50]. Notably, the DCHS has previously reported, on the basis of sensitivity and specificity analyses, that the SRQ-20 is a valid screening tool both antenatally and postnatally in our setting [49]. Similar findings were obtained in a birth cohort study in Ethiopia (P-MaMiE*)* [32,53].

#### 2.2.3 Other maternal psychosocial assessments

Various other psychosocial assessments were also collected between 28 and 32 weeks of gestation at an antenatal study visit. These measures included the Beck Depression Inventory (BDI), a validated and reliable screening tool for depressive symptoms [45,54,55], to asses maternal depression; the Intimate Partner Violence (IPV) Questionnaire, another widely used and validated measurement tool, to assess maternal physical, emotional and sexual violence exposure [56,57]. Exposure to IPV was defined as experiencing multiple events in any of these three exposure categories in the 12 months prior to the study visit; the Childhood Trauma Questionnaire – Short Form to assess childhood abuse and neglect [45,58]. The Modified Post-traumatic Stress Disorder Symptom Scale was used screen for post-traumatic stress disorder (PTSD) [59] and the Alcohol, Smoking and Substance Involvement Screen Test (ASSIST) was used to assess tobacco and alcohol use during the past three months [60]. The scoring and use of all these measures have been previously described [42].

Care was taken to ensure confidentiality and privacy during the interviews given the sensitive nature of some of the questions. In addition, if IPV was disclosed or any mental health issues were identified at the scheduled visits, staff referred participants to appropriate care or social services (including support services for IPV, substance abuse and mental disorders). Further, all women involved in the study received information regarding social and support service providers.

#### 2.2.4 Planning of pregnancy and partner support

The Planning of the Birth/Partner Support Questionnaire was used to assess pregnancy intention and support received from a male partner. Partner support for the pregnancy and ability to rely on a male partner for help are assessed on a Likert scale ranging from 1 (“not at all”) to 5 (“extremely”), with higher scores indicating greater support and partner reliability [52]. For the purposes of the study a three-level categorical variable was created to represent partner support of the pregnancy (“No/little support”, “moderate support”, and “high support”).

#### 2.2.5 Infant birth and development outcomes

Ultrasound measurements in the second trimester of pregnancy were used to measure gestational age. If ultrasound measurements were not available, the expected date of delivery was calculated using fundal height, which was recorded by trained clinical staff at enrolment, or from the last date of menstrual period [45]. Preterm birth was defined as a gestational age at delivery of less than 37 weeks.

Infant birth outcomes, including birthweight and head circumference, were measured at a single hospital where all the pregnancies in the area were delivered. Weight-for-age*-z-*score (WAZ) and head circumference-for-age*-z-*score (HCAZ) at birth were calculated using the revised Fenton growth charts [45,61,62]. Using these standards, infants were categorised as small for gestational-age (SGA) or appropriate/large (AGA/LGA) for gestational-age [45].

The infant developmental outcomes were measured using the Bayley III Scales of Infant and Toddler Development (BSID III), a well-validated developmental tool [63,64]. Children were assessed in five key developmental domains of cognition, language, motor, socio-emotional and adaptive behaviour. The BSID III is an individually-administered instrument designed to assess the developmental functioning of infants from birth to 24 months of age and it was administered by trained assessors blinded to child risk factors [63,65]. In the context of this study, the raw scores and composite scores, which correct for prematurity, were considered to describe the developmental domains at 6 months of age. The composite scores are generated using normative data for cognitive, language and motor domains and are scaled to have a mean of 100 and standard deviation of 15. It was decided to report the composite scores, as a similar trend between the raw scores and composite scores in the regression analysis were observed. The composite scores also allow comparability across domains. In addition, this study excluded the socio-emotional and adaptive behaviour domains, as these are based on limited assessment at such a young age. The administrations and calculation of scores has been previously describe in a DCHS study [66,67].

### 2.3 Data analysis

The analyses were conducted using STATA 14 (StataCorp Inc., College Station, Texas, USA).

Differences in binary/categorical characteristics between the recruitment site were identified using the Pearson’s Chi-square test of independence or Fisher’s Exact test (when observations were infrequent). In addition, the Wilcoxon Rank Sum test was used to test for differences in the continuous variables against recruitment site. Logistic regression was used to compute odds ratios (ORs) with 95% confidence intervals (CI) to determine the strength of the associations between psychological distress and sociodemographic, as well other psychosocial risk factors. In addition, a multivariable logistic regression model, including all predictors and/or psychosocial risk factor co-morbidities, was used to explore independent predictors/risk factors of antenatal psychological distress. The association between antenatal psychological distress and infant birth outcomes (preterm birth represented by gestational age, WAZ and HCAZ) were then explored in linear regression models. Covariates with p-values <0.2 were included in the multiple linear regression models as potential confounders.

Linear regression models were used to investigate the relationship between the developmental domains and antenatal psychological distress. Similar to the infant birth outcome models, the association between antenatal psychological distress, potential confounders, and infant development outcomes were then explored in multivariable linear regression models. Covariates with p-values <0.2 were considered in the multiple regression models. WAZ and HCAZ, were considered as covariates in the developmental outcome multiple linear regression models, although, only HCAZ was included as birth outcomes were highly correlated. Preterm birth was not considered as a covariate in these models, as the composite scores generated by the BSID III adjust for gestational age.

Diagnostic model checks were also performed on the multivariable models. Normality of residuals from the linear regression models were considered through histograms and quantile-quantile (Q-Q) plots, as well as the Shapiro-Wilk test. Homogeneity of variance of the residuals was investigated using Cameron and Trivedi's decomposition of IM-test, and the Breusch-Pagan/Cook-Weisberg test for heteroskedasticity. Further, the presence of multicollinearity was checked using variance inflation factor (VIF).

Interactions between the substance abuse variables and psychological distress were also explored, however these interactions were not statistically significant in the multiple regression models, nor did they improve the model, and thus were not reported.

Although use of dichotomised cut-offs on maternal psychosocial assessments may reduce statistical power, they are often used in clinical practice. Furthermore, on analysis of continuous maternal measures, similar findings were obtained.

## 3 Results

### 3.1 Demographic characteristics

A total of 1225 pregnant women were initially enrolled in the DCHS. However, 66 (6%) women were lost to follow-up between enrolment and delivery and 22 (2%) experienced pregnancy losses, including miscarriages, and still births. In addition, 176 (15%) women had incomplete data at the antenatal visit or delivery and were excluded from analysis. Thus, the sample utilised for this study included 961 women. The women lost to either follow-up or excluded from the analysis did not significantly differ on any key sociodemographic variables from the mothers who were included in this analysis. Similarly, those that did not complete the 6-month developmental measure did not significantly differ on demographic characteristics compared to those that did complete the measures, however tobacco and alcohol use, maternal IPV, and maternal depression were significantly different between the two groups. This was due to a higher frequency of alcohol and tobacco use in those that did not complete the 6-month developmental measure relative to those that did complete the BSID III. In addition, there was a higher frequency of mothers above threshold with regards to depressive symptoms and IPV exposure in the group that did not complete BSID III.

Table 1 presents baseline maternal demographic and psychosocial characteristics, stratified by recruitment site. The mean age of participants was 26 years (SD = 5.70). This sample included 40% women who were either married or cohabiting; 38% had completed secondary education or higher; 27% reported current employment; 37% reporting an average household income of less than R1000 per month (70 USD). The majority of women indicated that the current pregnancy was unplanned (66%). In addition, 19% of the women reported depressive symptoms; 34% reported child trauma and/or IPV exposure in the past 12 months; while 13% of the women were suspected of PTSD and 21% reported psychological distress symptoms. Demographic variables across the two recruitment sites were tabulated for descriptive purposes (Table 1). Women attending Mbekweni had lower levels of household income and a higher proportion of low SES groups, compared to mothers who attended TC Newman. Women from TC Newman experienced more trauma (on the CTQ and IPV) and PTSD, as well as higher frequency of substance abuse (tobacco and alcohol abuse), compared to those who attended Mbekweni.

**Table 1:** Maternal demographic and psychosocial characteristics and infant birth outcomes.

Variable	Mbekweni – n (%)	TC Newman – n (%)	Total – *n* (%)	*P*-value
Number of mothers	526 (54.73)	435 (45.27)	961 (100)	–
*Maternal Sociodemographic and psychosocial characteristics*				
Mean maternal age at enrolment (years) (SD)	27 (5.87)	25 (5.37)	26 (5.70)	<0.001
Ethnicity				
African-ancestry	520 (98.86)	5 (1.15)	525 (54.63)	<0.001
Mixed-ancestry	6 (1.14)	430 (98.85)	436 (45.37)	–
HIV positive	198 (37.29)	16 (3.70)	214 (22.22)	<0.001
Married/cohabiting	190 (36.12)	197 (45.29)	387 (40.27)	0.004
Partner/father is supportive of pregnancy				
No/little support	40 (7.63)	43 (9.91)	83 (8.66)	<0.001
Moderate support	321 (61.26)	97 (22.35)	418 (43.63)	–
High support	163 (31.11)	294 (67.74)	457 (47.70)	–
Educational achievement				
Primary education	44 (8.37)	35 (8.05)	79 (8.22)	0.091
Some secondary education	290 (55.13)	225 (51.72)	515 (53.59)	–
Completed secondary education	155 (29.47)	156 (35.86)	311 (32.36)	–
Tertiary education	37 (7.03)	19 (4.37)	56 (5.83)	–
Employed	123 (23.38)	132 (30.34)	255 (26.53)	0.015
Average household income				
<R1000/month^a^	231 (43.92)	126 (28.97)	357 (37.15)	<0.001
R1000-R5000/month	240 (45.63)	228 (52.41)	468 (48.70)	–
>R5000/month^b^	55 (10.46)	81 (18.62)	136 (14.15)	–
SES Quartiles				
Lowest SES	164 (31.18)	76 (17.47)	240 (24.97)	<0.001
Low-Mod SES	137 (26.05)	108 (24.83)	245 (25.49)	–
Mod-High SES	131 (24.90)	111 (25.52)	242 (25.18)	–
High SES	94 (17.87)	140 (32.18)	234 (24.35)	–
Unplanned pregnancy	357 (67.87)	274 (62.99)	631 (65.66)	0.113
Childhood trauma – above threshold (>36)	149 (28.33)	181 (41.61)	330 (34.34)	<0.001
Past year intimate partner violence	148 (28.14)	177 (40.69)	325 (33.82)	<0.001
Trauma exposure (Broad categorisation)				
Trauma-exposed	68 (12.93)	54 (12.41)	122 (12.70)	<0.001
Suspected post-traumatic stress disorder	91 (17.30)	35 (8.05)	126 (13.11)	–
Mean psychological distress score (SD)	3.86 (3.62)	5.21 (3.95)	4.47 (3.83)	<0.001
Psychological distress – above threshold (>8)	90 (17.11)	107 (24.60)	197 (20.50)	0.004
BDI – above threshold (>20)	92 (17.49)	89 (20.46)	181 (18.83)	0.241
Antenatal smoking (any use)	28 (5.32)	237 (54.48)	265 (27.58)	<0.001
Antenatal alcohol use (any use)	41 (7.79)	120 (27.59)	161 (16.75)	<0.01
				
*Infant birth outcomes*				
Number of infants; sets of twins; set triplets	531 (54.97); 3; 1	435 (45.03); 0; 0	966; 3; 1	
Male	262 (49.34)	238 (54.71)	500 (51.76)	0.096
Preterm (<37 weeks)	85 (16.01)	68 (15.63)	153 (15.84)	0.874
Mean Gestational age in weeks (SD)	38.50 (2.75)	38.35 (2.66)	38.43 (2.71)	0.245
Mean WAZ^c^ (SD)	−0.46 (1.08)	−0.73 (1.02)	−0.58 (1.06)	<0.001
Mean HCAZ^d^ (SD)	−0.38 (1.33)	−0.63 (1.17)	−0.49 (1.26)	0.006
Small for gestational age				
SGA	120 (22.77)	123 (28.28)	243 (25.26)	0.058
LGA	29 (5.50)	10 (2.30)	39 (4.05)	–
*Infant development at six months*				
Mean Cognitive domain score^e^ (SD; n)	102.28 (12.04; 112)	100.73 (12.90; 117)	101.48 (12.49; 229)	0.280
Mean Language domain score (SD; n)	105.02 (14.91; 110)	102.74 (15.21; 114)	103.86 (15.07; 224)	0.224
Mean Motor domain score (SD; n)	111.21 (13.84; 112)	110.22 (15.37; 117)	110.70 (14.62; 229)	0.680

a 1000 ZAR = 70 USD.

b 5000 ZAR = 340 USD.

c Weight-for-age z-score.

d Head circumference-for-age z-score.

e Composite scores.

### 3.2 Antenatal maternal psychological distress

Above threshold antenatal maternal psychological distress was observed in 197 (20.5%) women, Table 1. In crude analysis, those in this category were significantly more likely to be from the TC Newman recruitment site, and to score above threshold for antenatal tobacco use, antenatal alcohol use, childhood trauma, past-year IPV, depression, and PTSD (Table 2). Further, antenatal maternal psychological distress was less likely in mothers who completed secondary education compared to those who completed primary education, and in those whose partners were supportive of the pregnancy. In a multiple logistic regression model, adjusting for all variables in Table 2, antenatal maternal psychological distress remained associated with maternal childhood trauma (adjusted OR = 1.66, 95% CI: 1.14; 2.40), antenatal depression (adjusted OR = 7.56, 95% CI: 4.08; 11.26), and PTSD (adjusted OR = 1.87, 95% CI:1.12; 3.12) and inversely associated with high partner support (adjusted OR = 0.39, 95% CI: 0.21; 0.75).

**Table 2:** Variables associated with maternal psychological distress.

Variables	Above threshold ≥8 – n (%)	Unadjusted odds ratio (95% CI)	Adjusted odds ratio (95% CI), *n = 955*
***Demographic risk factors***			
Recruitment site			
TC Newman	107 (24.60)	Reference	Reference
Mbekweni	90 (17.11)	**0.63 (0.46, 0.87)**	**0.53 (0.32, 0.87)**
Marital status			
Married/cohabiting	78 (20.16)	Reference	Reference
Single	119 (20.73)	1.04 (0.75, 1.43)	0.78 (0.52, 1.17)
Partner/father is supportive of pregnancy			
No/little support	31 (37.35)	Reference	Reference
Moderate support	91 (21.77)	**0.47 (0.28, 0.77)**	078 (0.43, 1.44)
High support	74 (16.19)	**0.32 (0.19, 0.54)**	**0.39 (0.21, 0.75)**
HIV infected			
No	156 (20.83)	Reference	Reference
Yes	42 (19.63)	0.91 (0.62, 1.34)	1.05 (0.64, 1.70)
Educational achievement			
Primary	23 (29.11)	Reference	Reference
Some secondary	108 (20.89)	0.65 (0.38, 1.10)	0.94 (0.49, 1.79)
Completed secondary	58 (18.47)	**0.55 (0.31, 0.96)**	0.87 (0.39, 1.93)
Tertiary	9 (16.07)	0.47 (0.20, 1.10)	0.751 (0.24, 2.36)
Employment			
Employed	50 (19.61)	Reference	Reference
Unemployed	147 (20.82)	1.08 (0.75, 1.54)	0.91 (0.58, 1.44)
SES quartile			
Highest SES	41 (17.52)	Reference	Reference
Moderate-high SES	47 (19.42)	1.13 (0.71, 1.80)	1.02 (0.58, 1.7)
Low-moderate SES	52 (21.22)	1.27 (0.80, 2.00)	0.80 (0.41, 1.57)
Lowest SES	57 (23.75)	1.47 (0.94, 2.30)	0.915 (0.42, 1.94)
***Psychosocial risk factors***			
Pregnancy planning			
Planned pregnancy	66 (20.00)	Reference	Reference
Unplanned pregnancy	131 (20.76)	1.05 (0.75, 1.46)	0.74 (0.50, 1.11)
Antenatal smoking			
Below threshold	128 (18.39)	Reference	Reference
Above threshold	69 (26.04)	**1.56 (1.12, 2.18)**	0.93 (0.58, 1.51)
Antenatal alcohol use			
Below threshold	153 (19.13)	Reference	Reference
Above threshold	44 (27.33)	**1.59 (1.08, 2.35)**	1.13 (0.70, 1.85)
Childhood trauma			
Below threshold	94 (14.90)	Reference	Reference
Above threshold (>36)	103 (31.21)	**2.59 (1.88, 3.57)**	**1.66 (1.14, 2.40)**
Intermate partner violence			
No past-year violence	104 (16.35)	Reference	Reference
Past-year violence	93 (28.62)	**2.05 (1.49, 2.82)**	1.28 (0.87, 1.87)
Trauma exposure			
No trauma exposure	132 (18.51)	Reference	Reference
Trauma-exposed	30 (24.59)	1.44 (0.91, 2.26)	1.42 (0.85, 2.38)
Suspected post-traumatic stress disorder	25 (27.78)	**1.69 (1.10, 2.61)**	**1.87 (1.12, 3.12)**
Depression^a^			
Below threshold	97 (12.44)	Reference	Reference
Above threshold (>20)	100 (55.25)	**8.69 (6.05, 12.49)**	**7.56 (4.08, 11.26)**

a Beck Depression Inventory (BDI) used to measure antenatal depression.

### 3.3 Infant birth outcomes

From the 961 women included in this study, 966 infants (including three sets of twins, and one set of triplets) were born and153 (16%) preterm births occurred. Preterm births were not statistically different between the two recruitment sites. In addition, the infants were smaller on average than reference populations from HIC, with a mean WAZ of -0.58 (SD = 1.06) and HCAZ of -0.49 (SD = 1.26). Further, 25% (*n* = 243) of infants were classified as SGA. The four non-singleton births were included in this analysis as Fenton WAZ and HCAZ do take into account preterm birth and as non-singleton pregnancies are more likely to be preterm. Similar results were obtained if the non-singleton births were excluded. Thus, they were included to retain maximum statistical power.

#### 3.3.1 Associations between maternal psychosocial distress and infant birth outcomes

Antenatal maternal psychological distress was found to predict smaller head circumference at birth, as antenatal maternal psychological distress was associated with HCAZ in both the unadjusted (coefficient=−0.34, 95% CI: −0.54, −0.14, p-value = 0.001) and adjusted models (coefficient=−0.30, 95% CI: −0.49, −0.10, p-value = 0.004), Table 3. Further, an association was found between antenatal maternal psychological distress and WAZ in an unadjusted model (coefficient=−0.18, 95% CI: −0.35, −0.01, p-value = 0.035). However, this association fell away in the adjusted model, Table 3. Notably, antenatal maternal smoking and alcohol consumption was associated with lowered birth weight compared to those below threshold for these risk factors. No association was observed between antenatal maternal psychosocial distress and preterm birth (Table not shown).

**Table 3:** Association between maternal psychological distress and infant birth outcomes.

	**Birth weight Z-score (WAZ)**			**Head circumference Z-score (HCAZ)**		
Variable	Mean WAZ (SD)	Unadjusted regression coefficient (95% CI), p	Adjusted regression coefficient (95% CI)^a^^c^, p	Mean HCAZ (SD)	Unadjusted regression coefficient (95% CI), p	Adjusted regression coefficient (95% CI)^b^^c^, p
***Demographic risk factors***						
Recruitment site						
TC Newman	−0.73 (1.01)	Reference	Reference	−0.63 (1.17)	Reference	Reference
Mbekweni	−0.46 (1.08)	**0.27 (0.13, 0.40), <0.001**	0.13 (−0.05, 0.28), 0.184	−0.38 (1.33)	**0.25 (0.08, 0.41), 0.003**	0.06 (−0.15, 0.270), 0.588
Sex						
Female	−0.50 (1.09)	Reference	Reference	−0.52 (1.35)	Reference	
Male	−0.66 (1.03)	**−0.16 (-0.30, 0.03), 0.018**	**−0.14 (**−**0.27,** −**0.01), 0.040**	−0.46 (1.18)	0.06 (−0.11, 0.22), 0.499	
Educational achievement						
Primary	−0.78 (1.09)	Reference	Reference	−0.60 (1.12)	Reference	
Some secondary	−0.56 (1.07)	0.23 (−0.03, 0.48), 0.078	0.18 (−0.08; 0.43), 0.179	−0.49 (1.29)	0.11 (−0.19, 0.41), 0.463	
Completed secondary	−0.58 (1.01)	0.20 (−0.06, 0.46), 0.135	0.06 (−0.25; 0.36), 0.716	−0.47 (1.26)	0.12 (−0.19, 0.44), 0.440	
Tertiary education	−0.53 (1.14)	0.26 (−0.11, 0.62), 0.167	0.02 (-0.40; 0.43), 0.940	−0.51 (1.23)	0.09 (−0.35, 0.52), 0.697	
Employment						
Employed	−0.47 (1.05)	Reference	Reference	−0.41 (1.27)	Reference	Reference
Unemployed	−0.62 (1.05)	**−0.15 (-0.30, -0.00), 0.035**	−0.11 (−0.28, 0.05), 0.181	−0.52 (1.26)	−0.12 (−0.31, 0.06), 0.182	−0.14 (−0.3, 0.05), 0.158
SES quartile						
Highest	−0.48 (0.97)	Reference	Reference	−0.43 (1.23)	Reference	Reference
Moderate-high	−0.62 (1.11)	−0.15 (−0.34, 0.04), 0.132	−0.16 (−0.37, 0.05), 0.126	−0.58 (1.24)	−0.17 (−0.39, 0.06), 0.149	−0.14 (−0.37, 0.09), 0.240
Low-moderate	−0.64 (1.08)	−0.16 (−0.35, 0.03), 0.094	−0.18 (−0.42, −0.06), 0.133	−0.56 (1.28)	−0.13 (−0.36, 0.10), 0.258	−0.06 (−0.30, 0.17), 0.592
Lowest	−0.59 (1.08)	−0.12 (−0.31, 0.08), 0.238	−0.13 (−0.41, 0.14), 0.334	−0.39 (1.29)	0.04 (−0.19, 0.26), 0.761	0.10 (−0.15, 0.35), 0.440
HIV exposed						
No	−0.60 (1.05)	Reference		−0.52 (1.23)	Reference	Reference
Yes	−0.51 (1.10)	0.09 (−0.07, 0.26), 0.254		−0.38 (1.37)	0.14 (−0.05, 0.34), 0.150	0.04 (−0.17, 0.25), 0.729
***Psychosocial risk factors***						
*Antenatal smoking*						
Below threshold	−0.48 (1.09)	Reference	Reference	−0.40 (1.29)	Reference	Reference
Above threshold	−0.85 (0.93)	**−0.36 (**−**0.51,** −**0.22), <0.001**	**−0.20 (**−**0.38, -0.01), 0.038**	−0.74 (1.17)	**−0.34 (**−**0.51, -0.16), <0.001**	−0.16 (−0.38, 0.06), 0.164
*Antenatal alcohol use*						
Below threshold	−0.51 (1.06)	Reference	Reference	−0.41 (1.28)	Reference	Reference
Above threshold	−0.92 (1.00)	**−0.40 (**−**0.58, -0.22), <0.001**	**−0.26 (**−**0.45, -0.07), 0.007**	−0.88 (1.12)	**−0.46 (**−**0.68, -0.25), <0.001**	**−0.34 (**−**0.56. -0.11), 0.004**
*Intimate partner violence*						
No past-year violence	−0.52 (1.05)	Reference		−0.42 (1.30)	Reference	
Past-year violence	−0.70 (1.05)	**−0.18 (**−**0.32, -0.04), 0.014**		−0.64 (1.17)	**−0.22 (**−**0.39,** −**0.05), 0.012**	
*Trauma exposure*						
No trauma exposure	−0.58 (1.03)	Reference		−0.50 (1.25)	Reference	
Trauma-exposed	−0.67 (1.04)	−0.09 (−0.29, 0.11), 0.381		−0.58 (1.14)	−0.08 (−0.33, 0.16), 0.493	
Suspected PTSD	−0.52 (1.23)	0.06 (−0.14, 0.27), 0.548		−0.38 (1.46)	0.12 (−0.12, 0.36), 0.326	
*Depression*						
Below threshold	−0.54 (1.06)			−0.43 (1.28)	Reference	
Above threshold	−0.75 (1.02)	**−0.21 (**−**0.38, -0.04), 0.015**		−0.75 (1.15)	**−0.32 (**−**0.52,** −**0.11), 0.002**	
***Psychological distress***						
**Below threshold**	−0.54 (1.07)	Reference	Reference	−0.42 (1.29)	Reference	Reference
**Above threshold**	−0.73 (0.99)	**−0.18 (**−**0.35,** −**0.01), 0.035**	−0.13 (−0.29, 0.04), 0.129	−0.76 (1.13)	**−0.34 (**−**0.54, -0.14), 0.001**	**−0.30 (**−**0.49,** −**0.10), 0.004**

NOTE: Psychosocial risk factors (Intimate partner violence, trauma, depression) excluded from multiple models as highly associated with psychological distress.

a WAZ multiple regression model n = 959.

b HCAZ multiple regression model n = 949.

c Covariates with p-value <0.2 included in multiple linear regression models.

### 3.4 Infant developmental outcomes at 6 months

A total of 231 infants were included in the analysis. Detailed developmental outcomes at six months are presented in Table 1. Statistical comparisons showed that the mean scores for the cognitive, language and motor domains were similar across the two recruitment sites.

#### 3.4.1 Association between maternal psychosocial distress and infant development outcomes at 6 months

##### 3.4.1.1 Cognitive domain

In unadjusted analysis, cases with above threshold tobacco use compared to below threshold tobacco use differed significantly with the former having a significantly lower mean cognitive domain score. Similarly, those exposed to above threshold antenatal maternal depression had a significantly lower composite cognitive domain mean score, compared to those unexposed to maternal depression, Table 4. A higher composite cognitive domain score was associated with secondary and tertiary maternal education relative to primary maternal education. In addition, WAZ and HCAZ were also found to have a positive association with the composite cognitive domain score, Table 4. However, no association was observed between above threshold psychological distress and the BSID III Cognitive domain score in the adjusted multiple regression model.

**Table 4:** Association between maternal psychosocial distress and infant developmental outcomes at six months.

	Cognitive domain			Language domain			Motor domain		
Variable	Mean score (SD; n)	Unadjusted regression coefficient (95% CI), p	Adjusted Regression coefficient (95% CI)^a^, p	Mean score (SD; n)	Unadjusted regression coefficient (95% CI), p	Adjusted Regression coefficient (95% CI)^a^, p	Mean score (SD; n)	Unadjusted regression coefficient (95% CI), p	Adjusted Regression coefficient (95% CI)^#^, p
*Recruitment site*									
TC Newman	100.7 (12.9; 117)	Reference		102.7 (15.2; 114)	Reference		110.2 (15.3; 117)	Reference	
Mbekweni	102.3 (12.0; 112)	1.55 -1.70, 4.80), 0.349		105.0 (14.9; 110)	2.28 (−1.69, 6.25), 0.258		111.2(13.8; 112)	0.98 (−2.83, 4.80), 0.612	
Sex									
Female	101.7(12.9; 109)	Reference		105.2 (15.1; 106)	Reference		111.6 (15.2; 109)	Reference	
Male	101.3 (12.1; 120)	−0.46 (−3.72, 2.81), 0.782		102.7 (15.0; 118)	−2.47 (−6.44, 1.50), 0.221		109.9 (14.1; 120)	−1.65 (−5.47, 2.16), 0.394	
*Education achievement*									
Primary	93.5 (16.5; 13)	Reference	Reference	95.38 (18.45; 13)	Reference	Reference	97.6 (20.9; 13)	Reference	Reference
Some secondary	102.1 (11.2; 133)	**8.68 (1.65, 15.72), 0.016**	**8.40 (1.46, 15.34), 0.018**	103.3 (15.4; 131)	7.90 (−0.62, 16.41), 0.069	5.94 (−2.85,14.72), 0.184	110.8 (13.2; 133)	**13.21 (5.05, 21.37), 0.002**	**12.89 (4.90, 20.87), 0.002**
Completed secondary	100.6 (12.4; 72)	7.09 (−0.20,14.39), 0.057	6.15 (−1.06, 13.37), 0.115	105.0 (13.8; 69)	**9.62 (0.76, 18.47), 0.033**	4.06 (−6.11,14.22), 0.432	111.6 (14.5; 72)	**13.98 (5.52, 22.44), 0.001**	**13.18 (4.88, 21.48), 0.002**
Any tertiary	109.1 (8.6; 11)	**15.63 (5.71, 25.55), 0.002**	**13.49 (3.56, 23.42), 0.008**	113.6 (9.1; 11)	**18.16 (6.17, 30.15), 0.003**	11.22 (−2.32, 24.75), 0.104	118.8 (15.9; 11)	**21.20 (9.70, 32.71), <0.001**	**19.00 (7.63, 30.37), 0.001**
*SES quartile*									
Highest	102.6 (11.5; 46)	Reference		107.2 (13.4; 44)	Reference	Reference	111.6 (16.9; 46)	Reference	
Moderate-high	103.6 (12.3; 64)	−1.01 (−3.74, 5.75), 0.677		106.5 (14.4; 64)	−0.75 (−6.50, 4.99), 0.797	0.60 (-5.43, 6.63), 0.844	113.4 (13.4; 64)	1.87 (−3.68, 7.42), 0.507	
Low-moderate	99.7 (11.7; 53)	−2.84 (−7.84, 2.15), 0.263		102.4 (15.4; 52)	−4.82 (−10.83, 1.19), 0.115	−3.36 (−10.41, 3.68), 0.348	109.8 (13.3; 53)	−1.81 (−7.59, 3.97), 538	
Lowest	100.1 (13.7; 66)	−2.48 (−7.23, 2.27), 0.304		100.2 (15.8; 64)	**−7.05 (−12.79,−1.30), 0.016**	−5.83 (−13.25, 1.59), 0.123	108.2 (14.9; 66)	−3.35 (−8.87, 2.16), 0.232	
*Birth outcomes*									
WAZ	–	**2.57 (1.02, 4.11), 0.001**		–	2.09 (0.16, 4.02), 0.034		–	**3.15 (1.33, 4.97), 0.001**	
HCAZ	–	**1.90(0.56, 3.23), 0.006**	**1.70 (0.33, 3.07), 0.015**	–	1.29(−0.37, 2.96), 0,128	0.91 (−0.80, 2.62), 0.296	–	**2.37 (0.81, 3.93), 0.003**	1.78 (0.21, 3.35), 0.027
*Antenatal smoking*									
Below threshold	103.0(12.5; 146)	Reference	Reference	105.4 (14.7; 144)	Reference	Reference	111.3 (14.5; 146)	Reference	
Above threshold	98.9 (12.2; 83)	**−4.12 (−7.47,−0.78), 0.016**	−3.28 (−6.81, 0.25), 0.069	101.1 (15.5; 80)	**−4.23 (−8.34,−0.12), 0.044**	−2.92 (−7.34, 1.50), 0.194	109.6 (14.8; 83)	−1.78 (−5.74, 2.18), 0.376	
*Antenatal alcohol use*									
Below threshold	102.1 (12.5; 179)	Reference	Reference	104.6 (15.2; 176)	Reference	Reference	112.3 (13.8; 179)	Reference	Reference
Above threshold	99.4 (12.2; 50)	−2.67 (−6.60, 1.26), 0.182	0.02 (−4.23, 3.98), 0.953	101.2 (14.4; 48)	−3.34 (−8.17, 1.48), 0.173	−1.22 (−6.34, 3.91), 0.640	105.0 (16.2; 50)	**−7.35 (−11.86,−2.83), 0.002**	**−5.83 (−10.35,−1.32),0.012**
*Intimate partner violence*									
No past-year violence	101.5 (11.6; 134)	Reference		104.3 (14.5; 131)	Reference		112.2 (13.0; 134)	Reference	
Past-year violence	101.4 (13.7; 95)	−0.11(−3.42, 3.20), 0.948		103.3 (15.9; 93)	−1.01 (−5.04, 3.03), 0.624		108.6 (16.5; 95)	−3.52 (-7.37, 0.32). 0.072	
*Trauma exposure*									
No trauma exposure	101.3 (12.9; 172)	Reference		104.4 (15.5; 167)	Reference		111.2 (15.1; 172)	Reference	
Trauma-exposed	99.8 (11.1; 30)	−1.44(−6.38, 3.49), 0.565		101.7 (12.4; 30)	−2.65(−8.55, 3.26), 0.378		110.5 (13.1; 30)	−0.69 (−6.40, 5.02), 0.811	
Suspected PTSD	104.6 (11.0; 27)	3.36 (−1.73, 8.45), 0.195		103.2 (15.6; 27)	−1.13 (−7.30, 5.05), 0.720		107.8 (13.2; 27)	−3.28(-9.35, 2.59), 0.266	
*Depression*									
Below threshold	102.5 (12.1; 172)	Reference		105.2 (14.9; 167)	Reference		111.6 (14.8; 172)	Reference	
Above threshold	98.5 (13.3; 57)	**−3.96 (−7.69,−0.23), 0.038**		99.9 (15.0; 57)	**−5.29 (−9.80,−0.78), 0.022**		107.9 (13.8; 57)	−3.74 (−8.12, 0.65), 0.094	
*Psychological distress*									
Below threshold	101.7 (12.7; 175)	Reference	Reference	103.9 (15.2; 171)	Reference	Reference	110.9 (14.6; 175)	Reference	Reference
Above threshold	100.7 (11.8; 54)	−1.09 (−4.93, 2.74), 0.574	0.29 (−3.54, 4.12), 0.882	103.7 (14.8; 53)	−0.21(−4.89, 4.47), 930	0.95 (−3.79, 5.69), 0.693	109.9 (14.8; 54)	−0.99 (−5.48, 3.50), 664	0.52 (−3.88, 4.91), 0.817

Cognitive domain multiple regression model n = 228; Language domain multiple regression model n = 223; Motor domain multiple regression model n = 228.

*Note*: Psychosocial risk factors (Intimate partner violence, trauma, depression) excluded from multiple models as highly associated with psychological distress; Multiple regression models do not adjust for WAZ, due to collinearity with HCAZ.

a Covariates with p-value <0.2 included in multiple linear regression models.

##### 3.4.1.2 Language domain

In unadjusted analysis, the lowest SES group compared to the highest SES group, differed significantly in terms of the BSID III composite language domain score, with those in the lowest SES having a significantly lower mean language domain score. Those exposed to above threshold antenatal tobacco use and above threshold maternal depression, also had significantly lower BSID III composite language mean scores compared to those below threshold for tobacco use and maternal depression respectively, Table 4. Similarly, for the maternal education level, secondary and tertiary maternal education respectively differed from the primary education in such a way that for both groups, the mean BSID III language mean scores were higher compared to the primary education group. In the adjusted model, no association was found between antenatal maternal psychological distress and the BSID III language composite score.

##### 3.4.1.3 Motor domain

In unadjusted analysis, a significantly lower BSID III Motor domain mean score was observed in those exposed to above threshold maternal alcohol use, relative to those unexposed. Both WAZ and HCAZ were positively associated with motor domain. In addition, some secondary, completed secondary and tertiary maternal education also had significantly higher composite motor domain mean scores compared to the primary education group, Table 4. In the multiple regression model, no association was found between psychological distress and the BSID III Motor domain score.

## 4 Discussion

In this peri-urban LMIC setting, a high prevalence of antenatal maternal psychological distress was observed, with one in ﬁve women (21%) having above threshold scores. This is consistent with a previous meta-analysis indicating a weighted mean depression prevalence of 11.3% and weighted mean anxiety prevalence of 14.8% in African settings [12], as well as with the reported weighted mean CMD prevalence of 15.6% in pregnant women across 13 studies conducted in LMIC settings [68]. These findings point to the potential value of screening for psychological distress during pregnancy in these contexts, and of targeted intervention.

In HIC, poor marital relationships, history of psychological disorders, poor social support and stressful life events have been found to be significant predictors of antenatal maternal psychological distress [[12], [13], [14], [15]]. The independent risk factors associated with antenatal maternal psychological distress in this sample included depression, maternal childhood trauma, post-traumatic stress disorder (PTSD), past-year IPV and antenatal maternal smoking and alcohol consumption. On the other hand, maternal education and partner support of the pregnancy were found to be associated with lower antenatal maternal psychological distress, a finding that has been previously found in other African settings [[16], [17], [18]]. In the adjusted model, maternal childhood trauma, PTSD and depression remained significantly associated with antenatal maternal psychological distress, and inversely associated with partner support, consistent with previous literature [69]. These associations suggest that traumatic life events, which are highly prevalent in LMIC settings [70] are particularly impactful in these contexts. Certainly, in the LMIC context, women are frequently faced with childhood trauma, gender-based violence, poverty, and restricted access to healthcare and support.

Despite the majority of births being either full term or late preterm, the mean WAZ and HCAZ were lower than that of the HIC reference category [61], with a high proportion (25%) of SGA infants. In addition, antenatal maternal psychological distress was found to be a risk factor for lower birth weight and smaller head circumference at birth in the crude analysis. However, the association between lower birth weight and antenatal maternal psychological distress fell away in the multiple regression model, a similar finding to that of the P-MaMiE [32]. The relationship between psychological distress and head circumference remained in the adjusted model. The observed association between antenatal maternal psychological distress and decreased HCAZ at birth, even after adjustment for potential confounders, is concerning, as smaller head circumference may have a negative impact on infant health and neurological development in later stages of life. These findings are consistent previous work demonstrating an association between antenatal maternal psychological distress and adverse birth outcomes [[22], [23], [24], [25],^30^,^31^,^45^]. These associations may be mediated through a range of psychobiological pathways; further research investigating speciﬁc mechanisms is needed.

Although an association between antenatal maternal psychological distress and birth outcomes was found, there was no association between antenatal maternal psychological distress and any of the developmental outcomes measured at 6 months of age. The lack of association between antenatal maternal psychological distress and the developmental outcomes at 6 months of age may reflect that the developmental outcome mean composite scores in this sample were within the normal range of standardised scores reported. In other words, the sampled population are meeting expected cognitive and neurological development standards at this stage of life. Alternatively, the sample size may not provide sufficient power to effectively examine the association of antenatal psychological distress with developmental outcomes. In addition, developmental assessment at 6 months of age, may not be adequately sensitive to identify more subtle effects on child development which may become manifest at older age points. Notably, however, in the P-MaMiE antenatal maternal psychological distress was also not associated with child cognitive, language or motor domains at 12 months of age [41].

A crude association between antenatal depression and the cognitive domain at 6 months of age was, however, observed. Other psychosocial risk factors, such as alcohol and tobacco use, which were also associated with antenatal maternal psychological distress in this study, were found to have a negative impact on the developmental domains. Birth outcomes (*i.e*. WAZ and HCAZ), were also associated with multiple developmental outcomes, thus antenatal maternal psychological distress may indirectly impact infant development through these associations. These findings are consistent with a previous study that found that smaller head circumference at birth was associated with suboptimal physical, neurological, and cognitive outcomes later in life [71]. Further research at older child age may further elucidate the impact that maternal psychosocial risk factors, and the interaction between these risk factors, may have on child development.

The longitudinal and prospective nature of the DCHS, with measurement of multiple risk factors as well as birth and developmental outcomes in a LMIC setting, provides a unique strength to this study. In addition, the sample is representative of a large proportion of the population in similar LMIC settings. Several limitations should however be emphasized. First, measures of psychological distress were self-reported. However, in previous work from the DCHS, we found high sensitivity (63%) and specificity (83%) for the cut-off score used. [49]. Second, the BSID III uses United States (US) population-based norms, and there are concerns about its reliability. However, this measure has been validated in Sub-Saharan African, including South Africa [64,72,73], Third, power may be reduced as maternal psychosocial risk factors were considered in a dichotomised form as opposed to continuous. However, the analysis was also performed with continuous antenatal maternal psychosocial risk factor scores and similar results were obtained. Further limitations included the small sample size related to the developmental outcomes and the possibility that unmeasured confounders may have contributed to our results.

## 5 Conclusion

The high levels of above threshold antenatal maternal psychological distress in our sample, its association with maternal child trauma, depression, and PTSD, its inverse association with partner support, and its association with negative birth outcomes all point to the potential value of screening for maternal psychological distress during pregnancy, and developing targeted interventions. Given the impact of antenatal maternal psychological distress on birth outcomes, further study of the relevant underlying mechanisms is warranted, and more research is needed to establish the longitudinal effects of psychological distress on later child health including long-term child-development outcomes.

## Contributors

All authors contributed to and have approved the final manuscript.

## Funding

The Drakenstein Child Health Study (DCHS) is funded by the Bill & Melinda Gates Foundation (OPP1017641); the South African Medical Research Council; National Research Foundation (105865), South Africa. WB is supported by the SAMRC National Health Scholars programme, CJW is supported by the Wellcome Trust through a Research Training Fellowship [203525/Z/16/Z], and SMK is funded by a MQ fellows award and EDCTP early-career fellowship.

## Declaration of Competing Interest

None.

## References

[1] Masse R. (2000). Qualitative and quantitative analyses of psychological distress: methodological complementarity and ontological incommensurability. Qual Health Res.

[2] Ridner S.H. (2004). Psychological distress: concept analysis. J Adv Nurs.

[3] Priest S., Austin M.-P., Barnett B., Buist A. (2008). A psychosocial risk assessment model (PRAM) for use with pregnant and postpartum women in primary care settings. Arch Womens Ment Health.

[4] Kingston D., McDonald S., Austin M.-P., Tough S. (2015). Association between prenatal and postnatal psychological distress and toddler cognitive development: a systematic review. PLoS One.

[5] Premji S. (2014). Perinatal distress in women in low- and middle-income countries: allostatic load as a framework to examine the effect of perinatal distress on preterm birth and infant health. Matern Child Health J.

[6] Abiodun O.A., Adetoro O.O., Ogunbode O.O. (1993). Psychiatric morbidity in a pregnant population in Nigeria. Gen Hosp Psychiatry.

[7] Aderibigbe Y., Gureje O. (1992). The validity of the 28-item general health questionnaire in a Nigerian antenatal clinic. Soc Psychiatry Psychiatr Epidemiol.

[8] Aderibigbe Y., Gureje O., Omigbodun O. (1993). Postnatal emotional disorders in Nigerian women: a study of antecedents and associations. Br J Psychiatry.

[9] Assael M., Namboze J., German G., Bennett F. (1972). Psychiatric disturbances during pregnancy in a rural group of African women. Soc Sci Med.

[10] Cox J. (1979). Psychiatric morbidity and pregnancy: a controlled study of 263 semi-rural Ugandan women. Br J Psychiatry.

[11] Nhiwatiwa S., Patel V., Acuda W. (1998). Predicting mental disorder with a screening questionnaire: a prospective cohort study from Zimbabwe. J Epidemiol Community Health.

[12] Sawyer A., Ayers S., Smith H. (2010). Pre- and postnatal psychological wellbeing in Africa: a systematic review. J Affect Disord.

[13] Beck C.T. (2001). Predictors of postpartum depression: an update. Nurs Res.

[14] O’Hara M., Swain A. (1996). Rates and risks of postpartum depression: a meta-analysis. Int Rev Psychiatry.

[15] Lusskin S., Pundiak T., Habib S. (2007). Perinatal depression: hiding in plain sight. Can J Psychiarty..

[16] Adewuya A.O., Ola B.A., Aloba O.O., Dada A.O., Fasoto O.O. (2007). Prevalence and correlates of depression in late pregnancy among Nigerian women. Depress Anxiety.

[17] Alami K.M., Kadri N., Berrada S. (2006). Prevalence and psychosocial correlates of depressed mood during pregnancy and after childbirth in a Moroccan sample. Arch Womens Ment Health.

[18] Esimai O.A., Fatoye F.O., Quiah A.G., Vidal O.E., Momoh R.M. (2008). Antepartum anxiety and depressive symptoms: a study of Nigerian women during the three trimesters of pregnancy. J Obstet Gynaecol (Lahore).

[19] Field T., Diego M., Hernandez-Reif M., Schanberg S., Kuhn C., Yando R. (2003). Pregnancy anxiety and comorbid depression and anger: effects on the fetus and neonate. Depress Anxiety.

[20] Andersson L., Sundström-Poromaa I., Wulff M., Aström M., Bixo M. (2004). Neonatal outcome following maternal antenatal depression and anxiety: a population-based study. Am J Epidemiol.

[21] Berle J., Mykletun A., Daltveit A., Rasmussen S., Holsten F., Dahl A. (2005). Neonatal outcomes in offspring of women with anxiety and depression during pregnancy. A linkage study from The Nord-Trøndelag Health Study (HUNT) and Medical Birth Registry of Norway. Arch Womens Ment Health.

[22] Dole N., Savitz D., Hertz-Picciotto I., Siega-Riz A., McMahon M., Buekens P. (2003). Maternal stress and preterm birth. Am J Epidemiol.

[23] Orr S., James S., Blackmore Prince C. (2002). Maternal prenatal depressive symptoms and spontaneous preterm births among African-American women in Baltimore, Maryland. Am J Epidemiol.

[24] Maina G., Saracco P., Giolito M., Danelon D., Bogetto F., Todros T. (2008). Impact of maternal psychological distress on fetal weight, prematurity and intrauterine growth retardation. J Affect Disord.

[25] Diego M., Jones N., Field T., Hernandez-Reif M., Schanberg S., Kuhn C. (2006). Gonzalez-Garcia A. Maternal Psychological Distress, Prenatal Cortisol, and Fetal Weight. Psychosom Med.

[26] Dunkel Schetter C., Tanner L. (2012). Anxiety, depression and stress in pregnancy: implications for mothers, children, research, and practice. Curr Opin Psychiatry.

[27] Kramer M., Lydon J., Séguin L., Goulet L., Kahn S., McNamara H. (2009). Stress pathways to spontaneous preterm birth: the role of stressors, psychological distress, and stress hormones. Am J Epidemiol.

[28] Dunkel Schetter C. (2011). Psychological science on pregnancy: stress processes, biopsychosocial models, and emerging research issues. Annu Rev Psychol.

[29] Lawn J., Gravett M., Nunes T., Rubens C., Stanton C. (2010). Global report on preterm birth and stillbirth (1 of 7): definitions, description of the burden and opportunities to improve data. BMC Pregn Childbirth.

[30] Rondó P., Ferreira R., Nogueira F., Ribeiro M., Lobert H., Artes R. (2003). Maternal psychological stress and distress as predictors of low birth weight, prematurity and intrauterine growth retardation. Eur J Clin Nutr.

[31] Nasreen H., Kabir Z., Forsell Y., Edhborg M. (2010). Low birth weight in offspring of women with depressive and anxiety symptoms during pregnancy: results from a population based study in Bangladesh. BMC Public Health.

[32] Hanlon C., Medhin G., Alem A., Tesfaye F., Lakew Z., Worku B. (2009). Impact of antenatal common mental disorders upon perinatal outcomes in Ethiopia: the P-MaMiE population-based cohort study. Trop Med Int Health.

[33] Glover V. (2014). Maternal depression, anxiety and stress during pregnancy and child outcome; what needs to be done. Best Pract Res Clin Obstet Gynaecol.

[34] Talge N., Neal C., Glover V. (2007). Antenatal maternal stress and long-term effects on child neurodevelopment: how and why?. J Child Psychol Psychiatry.

[35] Bergman K., Sarkar P., O’Connor T., Modi N., Glover V. (2007). Maternal stress during pregnancy predicts cognitive ability and fearfulness in infancy. J Am Acad Child Adolesc Psychiatry.

[36] Brouwers E., van Baar A., Pop V. (2001). Maternal anxiety during pregnancy and subsequent infant development. Infant Behav Dev.

[37] Laplante D., Barr R., Brunet A., Galbaud du Fort G., Meaney M., Saucier J. (2004). Stress during pregnancy affects general intellectual and language functioning in human toddlers. Pediatr Res.

[38] Laplante D., Zelazo P., Brunet A., King S. (2007). Functional play at 2 years of age: effects of prenatal maternal stress. Infancy.

[39] Zhu P., Sun M., Hao J., Chen Y., Jiang X., Tao R. (2014). Does prenatal maternal stress impair cognitive development and alter temperament characteristics in toddlers with healthy birth outcomes?. Dev Med Child Neurol.

[40] DiPietro J., Novak M., Costigan K., Atella L., Reusing S. (2006). Maternal psychological distress during pregnancy in relation to child development at age two. Child Dev.

[41] Servili C., Medhin G., Hanlon C., Tomlinson M., Worku B., Baheretibeb Y. (2010). Maternal common mental disorders and infant development in Ethiopia: the P-MaMiE Birth Cohort. BMC Public Health.

[42] Stein D.J.K.N., Donald K.A., Adnams C.M., Koopowitz S., Lund C., Marais A. (2015). Investigating the psychosocial determinants of child health in Africa: the drakenstein child health study. J Neurosci Methods.

[43] Black M., Walker S., Fernald L., Andersen C., DiGirolamo A., Lu C. (2017). Early childhood development coming of age: science through the life course. Lancet.

[44] Zar H., Barnett W., Myer L., Stein D., Nicol M. (2015). Investigating the early-life determinants of illness in Africa: the drakenstein child health study. Thorax.

[45] Brittain K., Myer L., Koen N., Koopowitz S., Donald K.A., Barnett W. (2015). Risk factors for antenatal depression and associations with infant birth outcomes: results from a south african birth cohort study. Paediatr Perinat Epidemiol.

[46] Myer L., Stein D., Grimsrud A., Seedat S., Williams D. (2008). Social determinants of psychological distress in a nationally-representative sample of South African adults. Soc Sci Med.

[47] Beusenberg M., Orley J. (1994). A user’s guide to the self reporting questionnaire (SRQ).

[48] Ventevogel P., De Vries G., Scholte W., Shinwari N., Faiz H., Nassery R. (2007). Properties of the Hopkins Symptom Checklist-25 (HSCL-25) and the Self-Reporting Questionnaire (SRQ-20) as screening instruments used in primary care in Afghanistan. Soc Psychiatry Psychiatr Epidemiol.

[49] van der Westhuizen C., Brittain K., Koen N., Maré K., Zar H., Stein D. (2018). Sensitivity and specificity of the SRQ-20 and the EPDS in diagnosing major depression ante- and postnatally in a south african birth cohort study. Int J Ment Health Addiction..

[50] Harpham T., Reichenheim M., Oser R., Thomas E., Hamid N., Jaswal S. (2003). Measuring mental health in a cost-effective manner. Health Policy Plan.

[51] Rumble S., Swartz L., Parry C., Zwarenstein M. (1996). Prevalence of psychiatric morbidity in the adult population of a rural South African village. Psychol Med (Paris).

[52] Stein D., Koen N., Donald K., Adnams C., Koopowitz S., Lund C. (2015). Investigating the psychosocial determinants of child health in Africa: the drakenstein child health study. J Neurosci Methods.

[53] Hanlon C., Medhin G., Alem A., Araya M., Abdulahi A., Hughes M. (2008). Detecting perinatal common mental disorders in Ethiopia: validation of the self-reporting questionnaire and Edinburgh Postnatal Depression Scale. J Affect Disord.

[54] Beck A., Ward C., Mendelson M., Mock J., Erbaugh J. (1961). An inventory for measuring depression. Arch Gen Psychiatry.

[55] Beck A., Steer R. (1984). Internal consistencies of the original and revised Beck Depression Inventory. J Clin Psychol.

[56] Jewkes R. (2002). Intimate partner violence: causes and prevention. Lancet.

[57] Shamu S., Abrahams N., Temmerman M., Musekiwa A., Zarowsky C. (2011). A systematic review of African studies on intimate partner violence against pregnant women: prevalence and risk factors. PLoS One.

[58] Bernstein D., Fink L., Handelsman L., Foote J., Lovejoy M., Wenzel K. (1994). Initial reliability and validity of a new retrospective measure of child abuse and neglect. Am J Psychiatry.

[59] Foa E., Riggs D., Dancu C., Rothbaum B. (1993). Reliability and validity of a brief instrument for assessing post-traumatic stress disorder. J Trauma Stress.

[60] (2002). Group WAW. The alcohol, smoking and substance involvement screening test (ASSIST): development, reliability and feasibility. Addiction.

[61] Fenton T., Kim J. (2013). A systematic review and meta-analysis to revise the Fenton growth chart for preterm infants. BMC Pediatr.

[62] Fenton T., Nasser R., Eliasziw M., Kim J., Bilan D., Sauve R. (2013). Validating the weight gain of preterm infants between the reference growth curve of the fetus and the term infant. BMC Pediatr.

[63] Johnson S., Moore T., Marlow N. (2014). Using the Bayley- III to assess neurodevelopmental delay: which cut-off should be used?. Pediatr Res.

[64] Ballot D.E., Ramdin T., Rakotsoane D., Agaba F., Davies V.A., Chirwa T. (2017). Use of the bayley scales of infant and toddler development, third edition, to assess developmental outcome in infants and young children in an urban setting in South Africa. Int Sch Res Notices.

[65] Albers C., Grieve A. (2007). Review of Bayley Scales of infant and toddler development (Third editon). J Psychoeduc Assess.

[66] Koen N., Brittain K., Donald K., Barnett W., Koopowitz S., Maré K. (2017). Stein D. Maternal posttraumatic stress disorder and infant developmental outcomes in a south african birth cohort study. Psychol Trauma.

[67] Donald K., Hoogenhout M., du Plooy C., Wedderburn C., Nhapi R., Barnett W. (2018). Drakenstein Child Health Study (DCHS): investigating determinants of early child development and cognition. Bmj Paediatr Open.

[68] Fisher J., Cabral de Mello M., Patel V., Rahman A., Tran T., Holton S. (2012). Prevalence and determinants of common perinatal mental disorders in women in low- and lower-middle-income countries: a systematic review. Bull World Health Organ.

[69] Choi K., Sikkema K., Vythilingum B., Geerts L., Faure S., Watt M. (2017). Maternal childhood trauma, postpartum depression, and infant outcomes: avoidant affective processing as a potential mechanism. J Affect Disord.

[70] Benjet C., Bromet E., Karam E.G., Kessler R.C., McLaughlin K.A., Ruscio A.M. (2016). The epidemiology of traumatic event exposure worldwide: results from the World Mental Health Survey Consortium. Psychol Med (Paris).

[71] Gampel S., Nomura Y. (2014). Short and long-term effects of compromised birth weight, head circumference, and apgar scores on neuropsychological development. J Psychol Abnorm Child.

[72] Ballot D.E., Potterton J., Chirwa T., Hilburn N., Cooper P.A. (2012). Developmental outcome of very low birth weight infants in a developing country. BMC Pediatr.

[73] Hanlon C., Medhin G., Worku B., Tomlinson M., Alem A., Dewey M. (2016). Adapting the Bayley Scales of infant and toddler development in Ethiopia: evaluation of reliability and validity. Child Care Health Dev.

